# Clinical and Genetic Characteristics of Patients with Mild Hyperphenylalaninemia Identified by Newborn Screening Program in Japan

**DOI:** 10.3390/ijns7010017

**Published:** 2021-03-18

**Authors:** Shino Odagiri, Daijiro Kabata, Shogo Tomita, Satoshi Kudo, Tomoko Sakaguchi, Noriko Nakano, Kouji Yamamoto, Haruo Shintaku, Takashi Hamazaki

**Affiliations:** 1 Department of Pediatrics, Osaka City University Graduate School of Medicine, Osaka 545-8585, Japan; shino.taniguchi@gmail.com (S.O.); kudo-satoshi@jfe-eng.co.jp (S.K.); saka-tomo@med.osaka-cu.ac.jp (T.S.); nakano.noriko@med.osaka-cu.ac.jp (N.N.); 2 Department of Medical Statistics, Osaka City University Graduate School of Medicine, Osaka 545-8585, Japan; kabata.daijiro@med.osaka-cu.ac.jp (D.K.); ssfrfc3k@icloud.com (S.T.); 3 Department of Biostatistics, Yokohama City University School of Medicine, Yokohama 236-0004, Japan; kouji_y@yokohama-cu.ac.jp; 4 Donated Course “Disability Medicine and Regenerative Medicine”, Osaka City University Graduate School of Medicine, Osaka 545-8585, Japan; shintakuh@med.osaka-cu.ac.jp

**Keywords:** phenylketonuria, hyperphenylalaninemia, phenylalanine hydroxylase, genetic analysis, neonatal screening, genotype–phenotype correlation

## Abstract

Phenylketonuria (PKU) and hyperphenylalaninemia (HPA), both identified in newborn screening, are attributable to variants in *PAH*. Reportedly, the p.R53H(c.158G>A) variant is common in patients with HPA in East Asia. Here, we aimed to define the association between p.R53H and HPA phenotype, and study the long-term outcome of patients with HPA carrying p.R53H. We retrospectively reviewed the genotype in 370 patients detected by newborn screening, and identified the phenotype in 280 (117, HPA; 163, PKU). p.R413P(c.1238G>C) was the most frequently found (*n* = 117, 31.6%) variant, followed by *p.R53H* (*n* = 89, 24.1%). The odds ratio for heterozygous p.R53H to cause HPA was 48.3 (95% CI 19.410–120.004). Furthermore, we assessed the non-linear association between the phenylalanine (Phe) value and elapsed time using the follow-up data of the blood Phe levels of 73 patients with HPA carrying p.R53H. The predicted levels peaked at 161.9 μmol (95% CI 152.088–172.343) at 50–60 months of age and did not exceed 360 μmol/L during the 210-month long observation period. The findings suggest that patients with HPA, carrying p.R53H, do not need frequent Phe monitoring as against those with PKU. Our study provides convincing evidence to determine clinical management of patients detected through newborn screening in Japan.

## 1. Introduction

Phenylketonuria (PKU) and hyperphenylalaninemia (HPA) are autosomal recessive disorders characterized by the deficiency of hepatic phenylalanine hydroxylase (PAH) [1]. This enzyme is encoded by *PAH* located on chromosome 12q, and comprising 13 exons and 12 introns. The genotype–phenotype correlations in PKU have been demonstrated using predicted PAH activity, which is the average in vitro residual PAH activity of two alleles [2].

The severity of the disorder is diverse, ranging from HPA to classical PKU that is characterized by high blood phenylalanine (Phe) levels [3]. The European and US guidelines for PKU recommend studying its molecular genetics and accumulating the data about correlations between the genotype and clinical phenotype [4,5]. Furthermore, more than 1100 variants of *PAH* have been recorded in the locus-specific database PAHvdb (http://www.biopku.org/pah/, accessed on 1 March 2021). However, these data are mainly gathered from European patients. The frequency of *PAH* variants in Japanese and other East Asian populations is different from that in Europeans. Therefore, recording data on the genotype–phenotype correlation in Japanese patients with PKU is required.

In Japan, a nationwide newborn screening (NBS) for PKU was launched in 1977. The current screening cut-off level for Phe on dried blood spots is set >120 µmol/L. When patients with PKU and related disorders are identified via NBS, their clinical phenotypes are classified as follows: (1) tetrahydrobiopterin (BH4) deficiency is ruled out by pteridine analysis and measurement of dihydropteridine reductase activity, (2) PAH deficiency is classified by pretreatment-Phe levels, and (3) Patients with <60 μmol/L Phe level are diagnosed with HPA while those with >360 μmol/L are further classified as BH4-responsive mild PKU or classical PKU based on BH4 loading test.

According to the integrative Japanese Genome Variation Database (iJGVD, https://ijgvd.megabank.tohoku.ac.jp, accessed on 18 February 2021), the allele frequency of p.R53H in the general Japanese population was reported to be as high as 5% [6]. Several studies have reported that p.R53H is associated with HPA phenotype [3,4,7,8,9].On the contrary, a few reports exist that correlates PKU with p.R53H [9,10]. 

In this study, we retrospectively reviewed the genotype–phenotype correlation of 370 patients who were diagnosed with PKU or HPA through NBS in Japan. Consequently, we found that the p.R53H genotype was associated with HPA phenotype, but not with that of PKU. Further, 73 of the patients with HPA carrying the p.R53H variant displayed stable blood Phe levels without needs of permanent treatments during the long-term follow-up. Our findings are especially useful for determining clinical management of such patients in Japan and other East Asian countries.

## 2. Materials and Methods

### 2.1. Study Design and Participants

This study was approved by the Institutional Review Board of Osaka City University Graduate School of Medicine (Osaka, Japan) (#3687). Written informed consent for the genetic analyses was obtained from all patients or their parents/guardians. The study included 370 Japanese patients analyzed for *PAH* at our facility during January 1998–March 2017, and for secondary examinations of HPA by NBS. We retrospectively reviewed the medical records of the patients to analyze their phenotype and genotype characteristics. The criterion for PKU phenotype was blood Phe level >600 μmol/L, and that for HPA phenotype was 120–600 μmol/L without Phe-restriction diet. Furthermore, we analyzed the genotype–phenotype correlation with *p.R53H* and the follow-up data of blood Phe levels of patients with HPA carrying *p.R53H* from the retrieved medical records.

### 2.2. Statistical Analysis

To evaluate whether the p.R53H variant is the cause underlying the HPA phenotype, we performed multivariate logistic regression analysis in the PKU (including HPA) pediatric patients who underwent genetic analysis using the variable indicating presence or absence of the HPA phenotype as the outcome and the number of the p.R53H variants as the explanatory variable. This regression model was adjusted the following covariates; the presence of the p.R413P, p.R241C(c.721C>T), p.R111*(c.721C>T), IVS4-1G>A(c.442-1G>A), p.R243Q(c.728G>A), p.T278I(c.833C>T), p.R252W(c.754C>T), p.Ex6-96A>G(c.611A>G) and variants in the *PAH* gene and sex. The multiple imputation was conducted to impute the missing values for all variables.

Furthermore, in order to assess the change of Phe level over time, we performed non-linear regression analysis with the Huber–White robust sandwich estimator of variance–covariance matrix. The robust estimator considers dependence in repeated measures within a single patient. Additionally, non-linear restricted-cubic-spline was conducted to assess the non-linear association between Phe level and the elapsed time. In this mode, Phe levels were used with natural-log transformation to satisfy the assumption of normality of the error distribution. 

All statistical tests were performed two-sided with 5% significance level. The analyses were conducted using R (https://www.r-project.org/foundation/, accessed on 21 December 2020) (https://cran.r-project.org/, accessed on 21 December 2020).

## 3. Results

**Figure 1:**
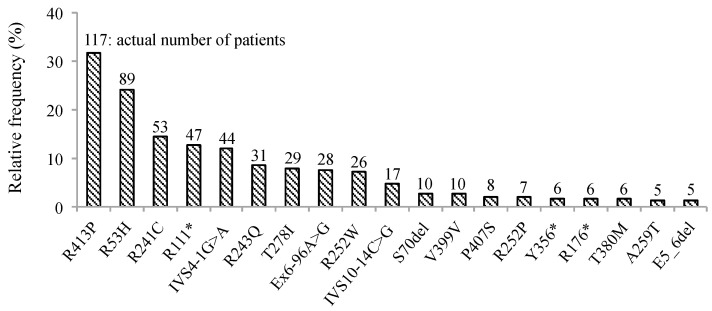
Frequency of *PAH* variants in 370 patients with elevated Phe levels detected by mass screening. Percentage of patients with mutations in *PAH* was detected by direct sequencing or multiplex ligation-dependent probe amplification (MLPA) in all the participants enrolled in this study. Numbers at the top of the vertical bars represent the actual number of patients with each mutation. Infrequent mutations have been omitted. Phe; phenylalanine, *PAH*; gene encoding phenylalanine hydroxylase, * stop codon.

We retrospectively examined the genotype and clinical phenotypic characteristics of the 370 subjects with PAH deficiency and blood Phe levels of >120 μmol/L detected through NBS. Frequency of variants in *PAH* observed are summarized in Figure 1, and all PAH variations (*n* = 370) found in the study cohort are shown in Table S1. Briefly, p.R413P was most frequently found (*n* = 117, 31.6%), followed by p.R53H (*n* = 89, 24.1%), p.R241C (*n* = 53, 14.3%), p.R111* (*n* = 47, 12.7%), and IVS4-1G>A (*n* = 44, 11.9%). Five patients had large deletions involving exons 5 and 6. In all, no variant was identified in three patients while one variant was identified in 27; two in 238; three in 11; and four in two. Phenotypes were identified in 280/370 patients, wherein HPA was identified in 117 and PKU in 163. 

**Figure 2:**
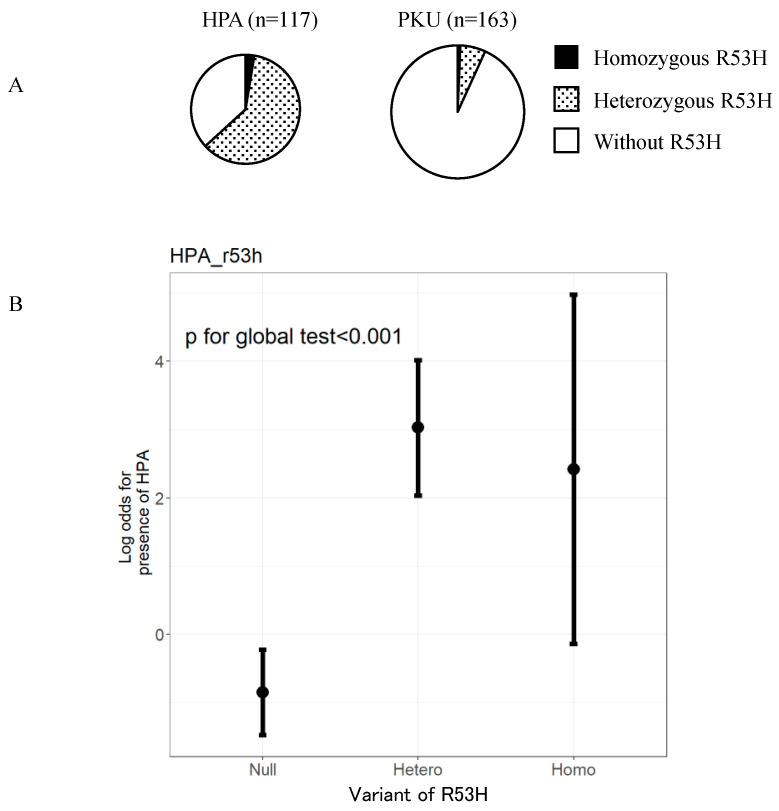
p.R53H variant is observed at a higher frequency in patients with HPA phenotype than in patients with PKU phenotype. (**A**) Number of patients with R53H variant in PAH (p.R53H) in each of the 280 patients with a known phenotype of either HPA or PKU. (**B**) Odds ratios for patients with no, heterozygous, and homozygous p.R53H variant to develop HPA phenotype. HPA, hyperphenylalaninemia; PKU, Phenylketonuria.

We, next, compared the frequency of the p.R53H variant between patients with HPA and PKU (Figure 2A). Consequently, we found that the frequency of p.R53H in those with HPA phenotype was 61.0% (74/117). From these, three were homozygous for p.R53H while 71 were heterozygous. The frequency of p.R53H in the patients with PKU phenotype was 6.7% (11/163). Of them, one was homozygous for p.R53H while 10 were heterozygous. In order to validate that the p.R53H variant is associated with HPA phenotype rather than that of PKU, we performed multivariate logistic regression analysis. Accordingly, we adjusted the influence of the other alleles frequently found in this population. The odds ratio for patients with heterozygous p.R53H to develop HPA phenotype was 48.3 (95% confidence interval [CI] 19.410–120.004) (Figure 2B). The odds ratio for patients with homozygous p.R53H to develop HPA phenotype was 26.2 (95% CI 2.054–334.832). This result demonstrated that patients with homozygous or heterozygous p.R53H variant are more likely to manifest HPA rather than PKU. 

We further examined the status of p.R53H in the 11 patients with PKU. Consequently, we found that one patient had four variants, homozygous p.R53H and homozygous p.R158W; seven patients had three variants, heterozygous p.R53H and two other variants, p.R413P/p.R252W, p.R111*/p.R252W, p.R111*/IVS9+1G>A(c.969+1G>A), IVS4-1G>A/p.R252W, p.R252W/p.EX6-96A>G, p.R252W/p.V399V(c.1197A>T), and p.P407S(c.1219C>T)/p.R158W(c.472C>T). Therefore, the presence of these variants other than *p.R53H* explains the manifestation of PKU in eight of the 11 identified patients [11,12,13,14,15,16,17,18]. The remaining three had only one heterozygous variant (p.R413P, p.R111*, and p.R252W) other than p.R53H.

To determine which of the genetic variants were responsible for HPA phenotype, we analyzed them in the 117 patients with HPA. We categorized the patients into three groups based on their phenotypes predicted from the previously reported genotype–phenotype correlations (Table 1). The left column lists 74 patients with HPA who carried the p.R53H variant. In these patients, genetic variants that are known to cause PKU, such as p.R413P and p.R243Q, were recurrently found. On the contrary, only three of 74 patients had the p.R241C variant, which was associated with BH4-responsive PKU. Patients homozygous for p.R53H were not found in our study. The middle column shows cases carrying variants associated with HPA except for p.R53H. The right column shows remaining cases carrying variants with predicted PKU or unknown phenotype. In this group, p.R241C variant was found in 18 patients. Three patients with homozygous p.R241C manifested the HPA phenotype, while only one patient with same the genotype manifested PKU phenotype (data not shown).

**Figure 3:**
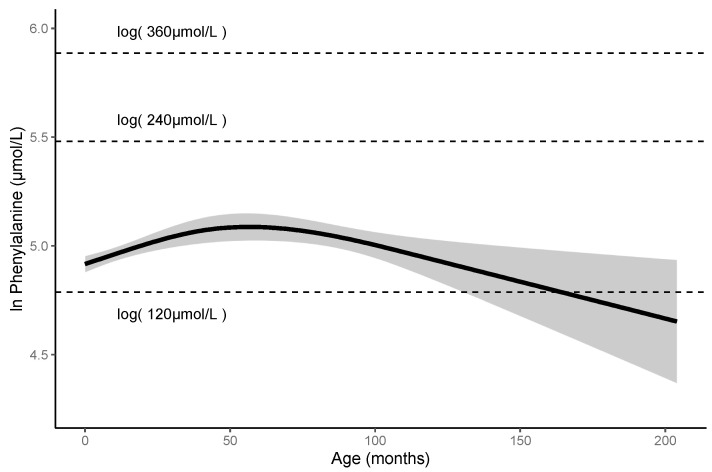
Predicted Phe levels until 200-month-olds are less than 360 μmol/L in patients with HPA carrying p.R53H.

There are no studies that report the long-term follow-up data of patients with HPA carrying p.R53H. We, thus, investigated the detailed clinicopathological characteristics of these patients. In the 74 patients with HPA carrying p.R53H, we excluded one patient carrying two pathogenic variants other than p.R53H. We analyzed the blood Phe levels in 73 patients (Figure 3). These patients included 31 males, 40 females, and two patients for whom gender data were not available. The observation period was of 0–210 months (median: 33 months). The mean of their blood Phe levels was 150 ± 30 μmol/L, and the maximum level found was 340 μmol/L. No patients had impaired mental and physical development. Furthermore, using the blood Phe levels at each visit of these patients and 635 data counts, we assessed the non-linear association between the Phe level and the elapsed time. The predicted Phe level at 0 month was 136.470 µmol/L (95% CI 131.491–141.638), but the Phe level peaked at 161.899 µmol/L (95% CI 152.088–172.343) at 50–60 months. Thereafter, the predicted Phe level gradually decreased to 106.246 μmol/L (95% CI 80.829–139.656) at 200 months of age. The predicted Phe levels did not exceed 360 μmol/L throughout the observation period. 

Changes in the predicted Phe levels in the patients. Solid line represents mean value; gray area represents 95% confidence interval. HPA, hyperphenylalaninemia; Phe; phenylalanine.

## 4. Discussion

We examined the genotypes of 370 patients with HPA or PKU identified by NBS in Japan. Consequently, we found that the p.R53H variant was recurrent in patients with HPA. Our study rigorously demonstrated that carrying compound heterozygous p.R53H and classical PKU-associated variants can predict HPA phenotype. Patients with PKU, carrying p.R53H, were likely to have two other pathogenic variants. Consequently, we found that the levels were maintained lower than 360 μmol/L until adolescence in the absence of Phe-restriction dietary treatment. Our findings provide convincing evidence that can help plan clinical management of patients detected through NBS in Japan. 

As shown in Figure 1, the most common variants identified in this study were p.R413P, p.R53H, and p.R241C. A few East Asian countries, namely Japan, China, Taiwan, and Korea, share a common spectrum of *PAH* variants [11,12,17,19,20]. The variants—p.R243Q, p.EX6-96A>G, p.R241C, and p.R413P—have been frequently detected in the these countries. Further, five variants—p.V399V, p.R111*, p.Y356X(c.1068C>A), IVS4-1G>A, and p.T278I—were shared with each other [12,17]. Allele frequency of p.R53H in the general population is 1.6% in the whole of East Asia, and 2.57% and 4.7% in Korea and Japan, respectively [21,22]. In case of patients detected through NBS, the allele frequency of p.R53H is 12.6% (93/740) in our study, and 1.27% and 2.11% in Korea and Taiwan, respectively [12,17]. Our study predicted the average Phe level for screening positive newborns carrying compound heterozygous p.R53H and other pathogenic variants to be 136.470 μmol/L (95% CI 131.491–141.638). In case of Korea and Taiwan, blood Phe levels of 240–599 μmol/L are used to define HPA; possibly, this is the reason why the p.R53H variant is less frequently detected in their affected populations [12,17]. The frequency of variants and genotypes in Japan is different from that recently published by Hillert et al. [23]. We considered that these differences were made because their study was based on information from only 55 patients. 

In this study, 6.7% patients with PKU carried p.R53H (allele frequency, approximately 4%) that is close to 4.7% in the Japanese general population, as mentioned above [21]. This fact also suggested that the p.R53H variant is not directly associated with PKU phenotype. In order to explain the pathogenesis of PKU, these 11 patients must have two other pathogenic variants in addition to p.R53H. However, we found that three of them carried only one other pathogenic variant. In these three patients we further performed multiple ligation-dependent probe amplification (MLPA) to exclude the possibility of large deletions. It should be noted that some genetic variants could not be identified even with the available methods. In previous studies, the detection rates of pathogenic variants among biochemically confirmed PKU cases were 96% (318/330) in China, 98% (139/142) in Taiwan, and 86.7% (137/158) in Korea [8,12,13]. Therefore, it is reasonable to assume that one other variant has not yet been identified in those patients who carry only one pathogenic variant other than p.R53H. 

In an analysis of 512 people in the Japanese general population, 48 people (allele frequency, 4.7%) were heterozygous for p.R53H [21]. These heterozygotes displayed an average 19% increase in plasma Phe levels compared to wildtype homozygotes. Interestingly, Choi in Korea reported that the father of a patient with HPA was homozygous for p.R53H but did not manifest HPA [22]. Consistent with these reports, in our study, patients homozygous for p.R53H were not detected by NBS. Our study revealed that patients with HPA and the p.R53H variant frequently carried compound heterozygous classical PKU-associated variant, such as p.R413P, p.R243Q, and p.T278I (Table 1). They were less likely to have the compound heterozygous mild PKU-associated variant, such as p.R241C. A study using COS cells has reported the residual enzyme activities of p.R413P, p.R53H, and p.R241C as 1%, 79%, and 49%, respectively [12]. Collectively, it is reasonable to assume that the combination of variants with high residual enzyme activity will not exceed 120 μmol/L Phe levels to be detected through NBS.

With respect to the cut-off value of Phe levels set for NBS, there are contradictory views; some argue that this cut-off value should be set higher because the current value detects people as PKU-affected even though they do not require treatment. However, patients with BH4 deficiency present around 120 μmol/L Phe levels [24]. Therefore, we consider that the current cut-off value should not be changed such that patients with BH4 deficiency who sometimes maintain low Phe levels are not missed during the screening process. Furthermore, DNAJC12 deficiency that manifests HPA phenotype but not *PAH* variant or BH4 deficiency has been reported earlier [25]. Further, patients with DNAJC12 deficiency who were diagnosed early and accordingly treated showed normal development, while permanent neurological damage was observed with delayed diagnosis. Since p.R53H is present at a high frequency in the Japanese general population [21], it is possible that patients with DNAJC12 deficiency carry this variant. When patients with p.R53H have persistently high Phe levels and exhibit neurological symptoms, such as developmental delay and behavioral disorder, DNAJC12 deficiency should be considered for differential diagnosis.

Since there are no guidelines for HPA in Japan, patients with this condition are also monitored according to the guidelines for PKU. The European guidelines for PKU recommend frequent measurements of blood Phe levels; weekly up to the age of 12 months, followed by fortnightly till 12 years, and monthly for >12 years [4]. The current Japanese follow-up guidelines of PKU recommend that patients undergo measurement of blood Phe levels every four weeks until elementary school entrance [26]. We found that the predicted Phe levels in patients with HPA carrying p.R53H never exceeded 360 μmol/L without Phe-restricted diet until 200 month of age (Figure 2). These facts suggest that patients with HPA do not require frequent follow-ups. Further, we recommend that guidelines be developed on a priority basis for patients with HPA. In patients with PKU, first trimester of pregnancy should be monitored since the Phe levels tend to elevate during this period [4]. According to the European and US guidelines, when the untreated blood Phe level of women of childbearing-age with PKU is 120–360 μmol/L, treatment is unnecessary [4,5]. Extrapolating our study findings, we expect the blood Phe levels of pregnant women with HPA carrying p.R53H to not exceed 360 μmol/L during pregnancy. Further, our findings suggest that Phe-restricted diet and frequent follow-ups are not necessary for such patients even during pregnancy.

There are several limitations associated with the present study. Owing to its retrospective nature, the protocol for genetic testing was not consistent from subject to subject. Only a subset of patients who were found to have one or fewer variants by direct sequencing were subjected to MLPA. In addition, for cases in which two or more pathogenic variants were identified by direct sequencing, no further analyses were performed, and the presence of large deletions may have been missed. Gülin Evinç et al. in Turkey report that children with untreated Phe levels of 240–360 μmol/L are at a higher risk for cognitive and behavioral impairment as compared to healthy children [27]. To evaluate neurodevelopmental outcome, appropriate developmental testing should have been performed in this study. 

Based on study findings, we propose the following measures when p.R53H is detected by genotype search about HPA of NBS in Japan. When identifying patients with PKU carrying p.R53H, presence of two other pathogenic variants should be looked for. Whereas, when patients with HPA carrying p.R53H are identified, *p.R53H* should be considered underlying the phenotype. This study revealed that blood Phe levels in patients with HPA carrying p.R53H were continuously below 360 μmol/L without diet therapy. However, we should not determine the follow-up frequency only by genotype. We suggest monthly follow-up at least in their infancy according to their diet change and increase in protein intake. The patients with persistently low Phe levels during infancy may not require the frequent hospital visits and the frequent measurements of blood Phe levels. In summary, we could clarify genotype–phenotype correlations and long-term follow-up data on blood Phe levels in patients with p.R53H and one genotype associated with PKU in the Japanese population. We therefore consider that the findings of our study hold implications in strategically planning appropriate follow-ups for patients with HPA. Future studies should investigate patient-oriented outcomes in the clinical management.

## Figures and Tables

**Table 1:** PAH variants identified in 117 patients with HPA phenotype.

Variants with R53H (*n* = 74)		Variants without R53H (*n* = 43)			
		Predicted HPA phenotype		Predicted PKU phenotype or unknown phenotype	
Genotype	*n*	Genotype	*n*	Genotype	*n*
R53H/R413P	15	R71H/T278I	1	R241C/R241C	3
R53H/R243Q	6	A132V/R413P	1	R241C/S70del	2
R53H/T278I	6	A132V/	1	R241C/P407S	1
R53H/Ex6-96A>G	5	R297C/ IVS4-1G>A	1	R241C/ IVS4-1G>A	1
R53H/IVS4-1G>A	4	A373T/R241C	1	R241C/Ex6-96A>G	1
R53H/R252W	4	A373T/T380M	1	R241C/R243Q	1
R53H/E5_6del	3	A373T/R413P	1	R241C/R252P	1
R53H/E3del4bp	3	Q375E/delS70	1	R241C/T278I	3
R53H/R111*	3	Q375E/ IVS4-1G>A	1	R241C/R413P	2
R53H/R241C	3	V379A/R111*	1	R241C/P281A	1
R53H/E6del	2	V379A/R252W	1	R241C/	2
R53H/V399V	2	T380M/IVS10-14C>G	3	R413P/	2
R53H/A259T	1	T380M/R111*	1	F55L/Y154D	1
R53H/S70del	1	T380M/ IVS4-1G>A	1	R243Q/	1
R53H/F402I	1	F402I/R252W	1	K431N/	1
R53H/IVS10-14C>G	1	A403V/S16*	1	S67C/	1
R53H/IVS10-1C>G	1	D415N/R353W	1		
R53H/L421T	1				
R53H/P281L	1				
R53H/R176X	1				
R53H/R243*	1				
R53H/R408W	1				
R53H/R413C	1				
R53H/V412P	1				
R53H/A132V/R413P	1				
R53H/	5				

## Data Availability

The data that support the findings of this study are available from the corresponding author, T.H., upon reasonable request.

## Supplementary Materials

The following are available online at https://www.mdpi.com/2409-515X/7/1/17/s1, Table S1: PAH variations (*n* = 370) found in the study cohort of PKU patients.
